# The Impact of Cd Pollution on Arbuscular Mycorrhizal Fungal Communities in Paddy Fields

**DOI:** 10.3390/plants14162501

**Published:** 2025-08-12

**Authors:** Wangbiao Xia, Yingchun Liao, Xinyi Chen, Liang Li, Yanning Shi, Yaxin Liu, Jingmin Zhang, Jiankang Fu

**Affiliations:** 1 School of Soil and Water Conservation, Jiangxi University of Water Resources and Electric Power, Nanchang 330099, China; wbxia0616@163.com (W.X.); y99_xinchen@163.com (X.C.); 13953828719@163.com (Y.S.); lyx03162022@163.com (Y.L.); 15679755487@163.com (J.Z.); 2 Institute of Microbiology, Jiangxi Academy of Sciences, Nanchang 330096, China; 3 College of Water Resources and Architectural Engineering, Northwest A&F University, Yangling 712100, China; fjk0919@foxmail.com

**Keywords:** arbuscular mycorrhizal fungi, cadmium pollution, paddy fields, soil parameters, community structure

## Abstract

Arbuscular mycorrhizal fungi (AMF) demonstrate considerable potential for remediating soils contaminated with heavy metals. However, comprehensive research examining the effects of cadmium (Cd) contamination on AMF communities in paddy fields remains scarce, constraining their broader application in such environments. In this study, high-throughput sequencing was utilized to assess AMF community structure in paddy soils subjected to five distinct levels of Cd contamination. The study also explored the effect of different soil properties on AMF community dynamics. A total of 188 AMF taxa were identified across all soil samples, spanning four families. The Claroideoglomeraceae family emerged as the predominant group, exhibiting notable Cd tolerance. While elevated Cd concentrations inhibited the AMF community structure, lower concentrations increased the α-diversity of the community. Furthermore, soil-available phosphorus, calcium levels, and pH were found to be critical factors driving shifts in AMF community structure. Redundancy analysis explicitly quantified the relative strength of environmental factors, demonstrating that phosphorus and pH directly influenced the AMF community structure through significant effects, while Cd and calcium exerted their influence via indirect or nonlinear pathways. Given the relative abundance advantage of Claroideoglomeraceae in Cd-contaminated environments and its positive correlation with Cd concentration, we hypothesize that this group may exhibit Cd tolerance. Therefore, it could be considered a potential candidate species for prioritization in future field inoculation trials, and its practical application potential should be further validated.

## 1. Introduction

Cadmium (Cd) is a highly toxic, non-biodegradable heavy metal that presents a serious threat to living organisms due to its high mobility in soil environments [1]. In China, the average concentration of Cd in agricultural soils is 0.19 mg/kg—approximately double the natural background level of 0.097 mg/kg. Alarmingly, Cd contamination affects 33.54% of farmland and 44.65% of urban soil sites [2]. Cadmium is readily absorbed by plants and can accrue in plants and enter the food chain, leading to biomagnification and potential health risks for humans. As a non-essential element for both plants and humans, Cd can inflict irreversible damage on crop productivity and human well-being [3,4]. Among numerous crops, rice demonstrates a particularly high capacity for Cd absorption and accumulation [5], potentially representing the primary pathway for human Cd exposure [6,7,8]. Considering the high consumption of rice in China, addressing food safety concerns and minimizing health risks is critical [6]. Therefore, ecological restoration of Cd-contaminated agricultural land has become an urgent priority. Traditional physicochemical remediation techniques, such as soil replacement and chemical leaching, can effectively reduce soil Cd content; however, they are often limited by high costs, disruption of soil structure, disturbance of microbial communities, and the risk of secondary pollution [9]. In contrast, bioremediation technology has emerged as a more attractive alternative due to its environmental friendliness, sustainability, and ecological compatibility. Specific microorganisms can reduce Cd bioavailability while preserving soil ecological functions. This ‘remediation while producing’ model is particularly suitable for contaminated farmland systems that require the maintenance of grain yield [10].

Arbuscular mycorrhizal fungi (AMF) exist ubiquitously across diverse ecosystems, surviving even in soils contaminated with heavy metals. These fungi establish symbiotic relationships with more than 80% of terrestrial plant species, enabling a bi-directional exchange of resources [11]. Through this partnership, host plants receive essential mineral nutrients—especially phosphorus—from AMF, while the fungi obtain carbohydrates necessary for their growth and survival from the host plants [12]. Additionally, AMF produces glomalin-related soil proteins (GRSPs), which function as chelating agents, binding soil heavy metals and reducing their bioavailability. This process offers protection to plants by alleviating the harmful impacts of heavy metal stress [13,14]. In the rice system, AMF reduce Cd uptake by roots and its translocation to grains by regulating the expression of Cd transport genes in rice, such as OsNramp5 and OsHMA2 [15]. Research indicates that following AMF inoculation, the Cd content in rice grains significantly decreases, while the biomass of rice increases by 40%. This finding is of great significance for ensuring the safe production of Cd-contaminated paddy fields [16]. Heavy metals also affect the diversity and spatial distribution of AMF communities [17], and in several field trials, these contaminants have been shown to significantly inhibit AMF activity [18,19]. However, systematic research on the impacts of Cd pollution on AMF community structure remains scarce in rice paddy systems. Existing studies predominantly focus on α-diversity metrics, failing to resolve response patterns of β-diversity to Cd gradients. Moreover, the regulatory interplay between soil factors (e.g., pH, available phosphorus, Ca) and Cd in shaping AMF community assembly has been neglected, with most investigations addressing solely Cd stress effects, which constrains the practical use of AMF-based microbial remediation strategies. Therefore, systematic investigation into how AMF community structures respond to Cd stress is essential for identifying Cd-tolerant AMF species and enhancing the development of effective bioremediation technologies.

This research aims to analyze AMF communities in Cd-contaminated paddy fields, contrasting with previous studies that primarily focused on laboratory settings. By examining Cd-affected soil in actual paddy field environments, we seek to elucidate the impact of Cd on AMF communities, identify dominant AMF species under Cd pollution, and investigate the primary environmental factors driving changes in AMF communities through a comprehensive analysis of soil physicochemical properties. The findings are anticipated to address the existing gap in dynamic data regarding AMF communities in paddy fields subjected to natural concentration gradients and to provide theoretical guidance for the application of AMF in the remediation of heavy metals in soil.

## 2. Results

### 2.1. Soil Physicochemical Properties

With the exception of alkali-hydrolyzable nitrogen, the other physicochemical properties of the five sampled plots demonstrated statistically significant differences (Table 1). Regarding Cd contamination, the XY_1 site exhibited a significant increase compared to the other four sites, with levels ranging from 102.99% to 277.78% higher. Based on China’s current “Soil Environmental Quality Standards” (GB15618-2018) [20], the Cd pollution at the XY_1 site is classified as moderate, whereas the NK site has the lowest Cd concentration. The soil organic matter and total nitrogen contents at the JX, WN, and XY_1 sites were significantly elevated compared to the NK site, showing increases between 35.88% and 64.35% for organic matter, and 47.37% to 55.56% for total nitrogen. The XY_1 and XY_5 plots also demonstrated significantly higher pH levels and calcium content compared to the JX, NK, and WN plots. Notably, among all plots, the NK plot recorded the lowest concentrations of organic matter, available phosphorus, total nitrogen, alkali-hydrolyzable nitrogen, and Cd content.

### 2.2. Analysis of AMF Community Structure and Diversity

**Figure 1:**
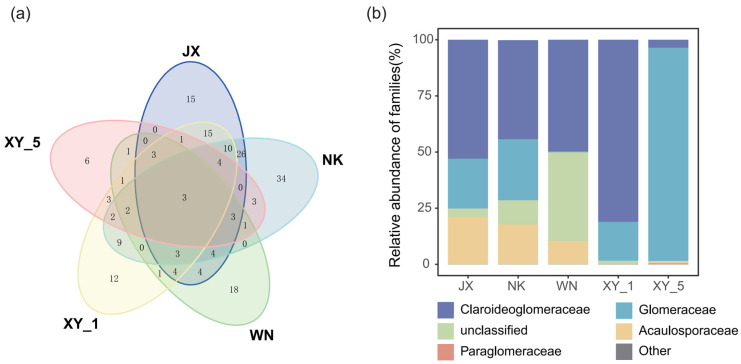
(**a**) Distribution Venn diagram of AMF OTU. The numbers represent the count of OTU unique to each sample plot, while the numbers in the overlapping sections indicate the count of OTU shared among different sample plots. (**b**) Analysis of AMF community composition at family levels. JX, Jinxi County; NK, Nankang District; WN, Wannian County; XY, Yushui District.

Using high-throughput sequencing technology, this study identified a total of 1,212,779 optimized amplified sequences and 188 AMF OTUs across five sample plots, encompassing one phylum, four classes, five orders, ten families, thirteen genera, and thirty-five species. The Venn diagram (Figure 1a) indicates that shared OTUs constituted 1.60% of the total. The NK plot demonstrated the highest number of unique OTUs, representing 18.09% of the total, while the XY_5 plot showed the lowest at 3.19%. In terms of relative abundance, the dominant families were as follows: Claroideoglomeraceae (46.29%), Glomeraceae (32.41%), unclassified families (11.08%), Acaulosporaceae (10.06%), Paraglomeraceae (0.12%), and others (0.04%) (Figure 1b). Among the groups, Glomeraceae (94.98%) dominated the XY_5 site, while Claroideoglomeraceae predominated in the remaining sites, comprising JX (53%), NK (44.09%), WN (49.71%), and XY_1 (81.08%). In contrast, the WN site showed a significant decline in Glomeraceae (0.42%) and a markedly higher proportion of unclassified families (39.47%) compared to the other groups. The AMF community compositions of the JX and NK sites demonstrated the highest similarity.

### 2.3. AMF Community Alpha Diversity Index

Significant variations in the α-diversity of AMF communities were observed across the five sampling plots, as shown in Table 2. The AMF richness was significantly higher in the JX and NK plots compared to the WN and XY_5 plots. Additionally, the NK plot exhibited a significantly higher Shannon index than both the WN and XY_5 sites. However, no significant differences were found in the Simpson index among the five groups. These findings indicate that the NK site harbors a more diverse AMF community. Overall, the α-diversity indices were relatively elevated in the JX and NK plots, moderate in the XY_1 plot, and comparatively low in the WN and XY_5 plots. Of all the plots, JX recorded the highest values across all four α-diversity indices.

### 2.4. AMF Community β-Diversity Index

**Figure 2:**
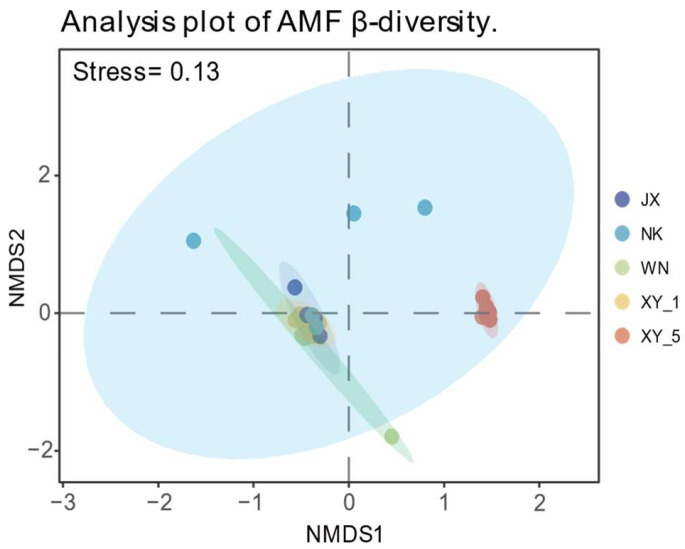
NMDS ordination shows the β-diversity of AMF community composition, with the ellipse representing the 95% confidence interval.

ANOSIM analysis revealed a statistically significant R-value of 0.507 (*p* = 0.001), indicating greater differences between groups than within groups. This suggests a clear separation trend among the groups, with relatively high similarity within groups and marked dissimilarity between them. Further interpretation of the NMDS ordination (Figure 2) revealed a widespread distribution of samples in the NK group, reflecting substantial variability in AMF community structure within this group. The 95% confidence intervals for the NK group overlapped with those of JX, XY_1, and XY_5, implying some compositional similarity among these groups. Conversely, samples in the XY_5 group were closely clustered, indicating a more uniform community structure, and were distinctly separated from the JX, XY_1, and WN samples, suggesting that the differences are statistically significant in the AMF community structure. In the WN group, one sample was notably distant from the other four, contributing to increased within-group variability.

### 2.5. Environmental Factors and AMF Communities

**Figure 3:**
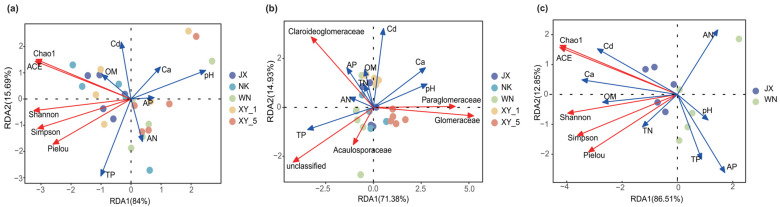
RDA of environmental factors and AMF diversity and family levels. (**a**) The correlation between AMF diversity and environmental factors. (**b**) The correlation between AMF and environmental factors at the family level. (**c**) The correlation between AMF and environmental factors at the family level in the JX and WN plots. The longer the arrow, the greater the impact of the factor; the smaller the angle between the two axes, the higher the correlation.

Redundancy analysis revealed that the Chao1, ACE, and Shannon indices were positively correlated with Cd, organic matter, and total phosphorus, but negatively correlated with calcium, available phosphorus, and pH. In contrast, the Simpson and Pielou indices showed positive correlations with organic matter, total phosphorus, and alkali-hydrolyzable nitrogen, while being negatively correlated with Cd, calcium, available phosphorus, and pH. Overall, the variation in AMF community distribution was explained by 99.69% of the total variance (Figure 3a), with total phosphorus (*p* = 0.147) emerging as the primary environmental factor influencing AMF community shifts. When analyzing the JX and WN plots separately, the RDA1 and RDA2 axes accounted for 86.51% and 12.85% of the variation, respectively (Figure 3c). In these plots, α-diversity indices were positively correlated with Cd, calcium, organic matter, and total nitrogen, but negatively correlated with available nitrogen and pH. Key environmental factors influencing AMF diversity and richness included Cd and calcium (*p* = 0.017), as well as available phosphorus (*p* = 0.034). At the family level, the first two RDA axes explained 71.38% and 14.93% of the total AMF community variation, respectively. Cd demonstrated positive correlations with Glomeraceae, Claroideoglomeraceae, and Paraglomeraceae families, and negative correlations with Acaulosporaceae and unclassified families. The analysis identified pH, total phosphorus, calcium (*p* ≤ 0.001), and Cd (*p* = 0.018) as the primary environmental factors influencing the relative abundance of AMF families (Figure 3c).

**Figure 4:**
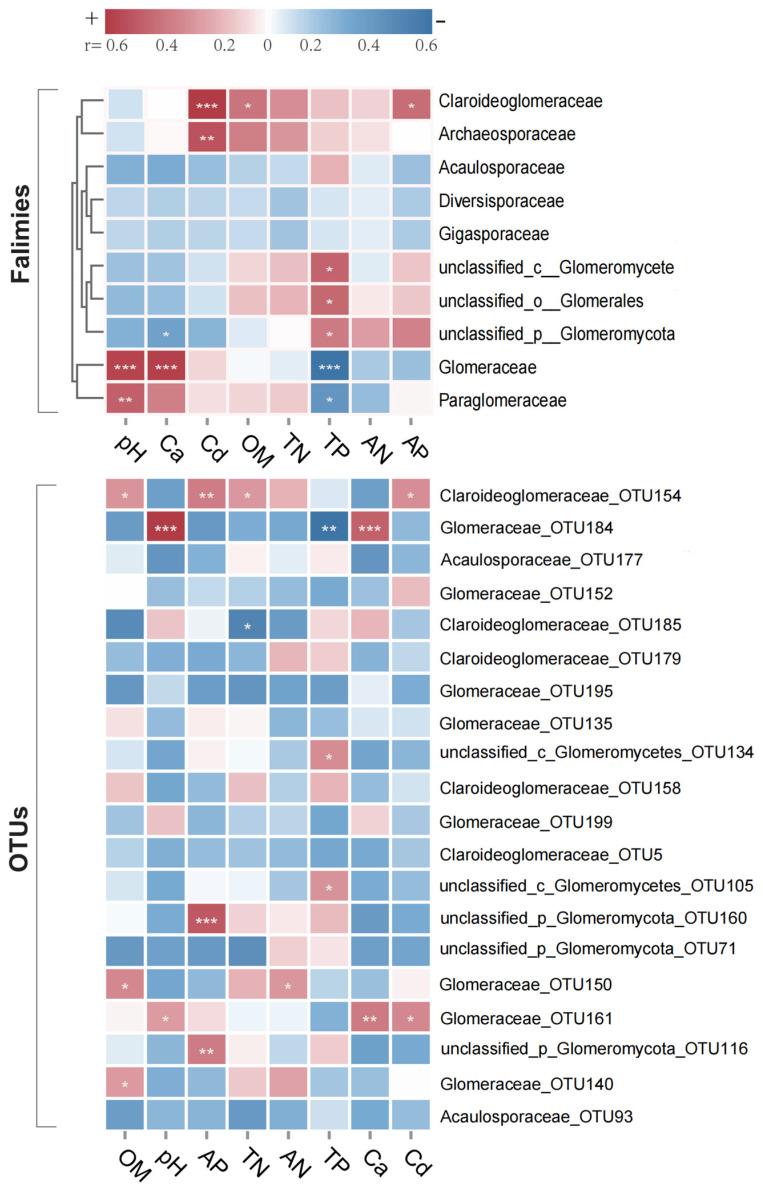
Heatmap of the correlation between soil physicochemical properties and dominant families and OTUs of AMF. The colors indicate the correlation coefficient (r). OM, organic matter; AP, available phosphorus. TN, total nitrogen; AN, alkali-hydrolyzed nitrogen; TP, total phosphorus; Ca, total calcium; Cd, total cadmium. * *p* < 0.05, ** *p* < 0.01, *** *p* < 0.001.

A heatmap was generated to visualize the correlation between environmental variables and AMF at the family level, along with the dominant OTUs, specifically the top 20 at the family level. Notably, Claroideoglomeraceae and Archaeosporaceae were clustered together, exhibiting a significant positive correlation with Cd (Figure 4). Furthermore, Glomeraceae and Paraglomeraceae demonstrated a significant positive correlation with soil pH, but a significant negative correlation with total phosphorus. Three unclassified AMF families formed a distinct cluster that was significantly positively correlated with total phosphorus, while an unclassified Glomeromycota family exhibited a significant negative correlation with calcium levels. Overall, the measured soil parameters had minimal influence on Acaulosporaceae, Diversisporaceae, and Gigasporaceae. Among all measured environmental variables, total nitrogen, and alkali-hydrolyzable nitrogen had relatively weak effects on AMF community structure at the family level, whereas total phosphorus had the most pronounced effect. At the taxonomic unit level, OTU154 and OTU161 showed significant positive correlations with Cd. The environmental factors most significantly correlated with OTU154 included Cd, total nitrogen, available phosphorus, and organic matter. Regarding environmental influences, soil organic matter, available phosphorus, and total phosphorus significantly affected three distinct OTUs, respectively. Other environmental variables, with the exception of alkali-hydrolyzable nitrogen, significantly influenced two OTUs each; alkali-hydrolyzable nitrogen significantly affected only OTU154.

## 3. Discussion

### 3.1. Dominant AMF Families in Cd-Contaminated Paddy Fields

This study reveals that Claroideoglomeraceae is the most prevalent AMF family in Cd-contaminated paddy soils, comprising 46.29% of the AMF community (Figure 1b). Previous research has demonstrated that Claroideoglomeraceae commonly colonize coastal regions [21] and are integral to a wide range of ecosystems, including orchards [22], marine environments [23], grasslands, and wheat fields [24]. The high diversity and adaptability of this fungus enable its widespread distribution across different environments, highlighting its potential for soil microbial remediation. This is particularly evident in agricultural lands heavily contaminated with heavy metals such as Cd, zinc, and lead, where the presence of Claroideoglomeraceae is notably high [19,25]. Previous studies suggest that the enhanced tolerance exhibited by Claroideoglomeraceae may be attributed to its secretion of GRSPs, which possesses chelating properties that can effectively bind and immobilize heavy metals. This function not only protects host plants from Cd toxicity but also minimizes potential risks to Glomeraceae itself [26]. Furthermore, the presence of metal transport proteins and detoxification genes in Claroideoglomeraceae may contribute to their ability to regulate heavy metal accumulation by actively expelling Cd from their cells [27]. However, it is noteworthy that a study conducted in an open-pit mining area indicated that the metal tolerance of the Claroideoglomeraceae family is relatively low [28]. This observation could be related to factors like varying metal concentrations and host plant species, indicating potential performance limitations under specific environmental conditions. The current study area exhibited high Glomeraceae abundance (20.21%), particularly at the XY_5 site. Research indicates that Glomeraceae’s characteristic mechanism of actively repairing damaged mycelium is essential for survival in adverse environments [29]. Both Claroideoglomeraceae and Paraglomeraceae demonstrate widespread distribution across different rice cultivars and in lowland paddy fields with significant variations in salinity and arsenic concentrations [30,31]. The widespread occurrence of these two AMF families within the study area underscore their potential for supporting the ecological restoration of Cd-contaminated paddy fields. Their symbiotic association with rice plants and ability to withstand environmental stressors render them valuable in mitigating heavy metal pollution in paddy fields.

### 3.2. Cd-Induced Changes in AMF Community Structure

A detailed examination of the AMF community structure across multiple sites revealed that the differences are statistically significant in both community structure and diversity (Figure 2, Table 2). The sites with low Cd concentrations displayed a more dispersed and uniform distribution pattern in the NMDS analysis, suggesting greater heterogeneity within the group. In contrast, sites with high Cd concentration exhibited more converged community structures. Further, the Chao1 and ACE indices demonstrated that the AMF species richness in low Cd-contaminated areas significantly exceeded that in high-Cd-contaminated areas. Similarly, the Shannon index indicated a more balanced AMF species community distribution under low Cd contamination. Collectively, these indicators corroborate previous findings of a negative correlation between soil Cd content and AMF diversity [18,32]. This negative correlation may stem from high Cd concentrations favoring competitive families such as Claroideoglomeraceae, while typically low-abundance families struggle to survive under Cd pollution, resulting in community structure simplification [33,34]. Notably, RDA revealed a seemingly contradictory pattern (Figure 3a,b)—a positive correlation between Cd levels and the α-diversity index. This observation might be explained by two potential mechanisms: first, low Cd concentrations may stimulate AMF taxa growth, with inhibition occurring above certain thresholds. Second, the relationship between Cd and α-diversity may be nonlinear. Additionally, interactions among environmental factors suggest that the positive correlation between Cd and α-diversity might partially reflect indirect effects of other variables. Although some existing studies support this hypothesis [27,35], future research should establish gradient Cd concentrations while controlling the interference of other variables to examine the direct impact of Cd on AMF communities.

Correlation analysis at the AMF family level (Figure 4) revealed varying relationships between AMF taxa and Cd in paddy fields. The family Claroideoglomeraceae showed broader distribution in Cd-contaminated paddy fields, a phenomenon particularly evident in samples from high-Cd areas. Glomeraceae abundance exceeded 30% in these regions, confirming its dominance in Cd-contaminated environments and providing a foundation for further research [27]. Multiple studies have demonstrated strong Cd tolerance in the Glomeraceae family [11,36], aligning with these observations. The notably low Glomeraceae abundance (0.42%) in the WN plot may result from growth inhibition by high available phosphorus in local soil [37]. Additionally, the exceptionally high Glomeraceae abundance in the XY_5 plot (94.98%) suggests that Cd tolerance may be significantly influenced by pH and calcium ion synergistic effects. While Paraglomeraceae also appeared to be promoted by Cd, its minimal abundance (0.12%) potentially indicates poor host plant adaptation, limiting its survival. Conversely, Acaulosporaceae demonstrated significant Cd inhibition, consistent with Zarei et al.’s findings [38]. Previous research indicates that even within identical AMF taxa categories, correlations with heavy metal contamination can vary [39]. Among the top 20 OTUs identified, 5 belonged to the Claroideoglomeraceae family. Of these, only one OTU showed a positive correlation with Cd, while the remaining four were negatively correlated (Figure 4), highlighting intra-family variability in Cd tolerance. Understanding the driving mechanisms and ecological effects of AMF community structure in Cd-affected environments remains crucial for developing ecological remediation systems based on the functional regulation of natural microorganisms.

### 3.3. Regulatory Mechanisms of Multiple Environmental Factors on AMF Communities

The redundancy analysis results revealed that Cd does not influence AMF communities in isolation. Instead, its effects are part of a complex interaction network involving soil parameters such as available phosphorus, calcium, and pH (Figure 3a,b). These factors work in concert to shape the AMF community dynamics [40]. Although our current study does not fully unravel the intrinsic mechanisms underlying these interactions, several significant associations have been identified. For example, a notable negative correlation was observed between Cd and available phosphorus, with AMF diversity significantly lower in areas characterized by low Cd and high phosphorus concentrations (Figure 3b, Table 2). This pattern may be attributed to the formation of insoluble Cd–phosphate compounds resulting from the interaction between Cd and phosphorus. When soil Cd concentrations exceed certain thresholds, these insoluble compounds reduce the overall available phosphorus in the soil. Moreover, high available phosphorus concentrations can diminish the symbiotic relationship between mycorrhizal fungi and host plants, as the dependency of plant roots on mycorrhizal fungi is contingent upon the supply of phosphorus [9,12]. A related study examining the combined effects of heavy metals and phosphorus in soil demonstrated that both AMF inoculation and phosphorus fertilizer application effectively reduce heavy metal stress on plants [41]. This discovery emphasizes the necessity of considering the integrated effects of heavy metals and other soil nutrients in management practices. Consequently, optimizing the balance between phosphate fertilizer application, AMF inoculation, and regulation of Cd concentration to create favorable conditions for rice growth while minimizing heavy metal impacts represents a crucial area for future research.

Our analysis of soil properties and AMF communities revealed complex interactions, particularly regarding soil Cd and phosphorus concentrations, along with correlations to soil pH levels and calcium content. Variance analysis highlighted several notable patterns (refer to Table 1 and Table 2). The observed variations in Cd and phosphorus concentrations across soil samples reflect both their chemical composition and potential influence on microbial communities. Acidic environments typically exhibit lower Cd and phosphorus concentrations, while alkaline conditions tend to show higher levels of these elements. This pH-dependent variation plays a critical role in shaping microbial growth and activity [28]. The study revealed that pH and calcium content significantly influence specific AMF families, particularly Glomeraceae. In soils with higher pH and calcium levels, Glomeraceae dominated, making up 94.98% of the AMF population, whereas, in other plots, Claroideoglomeraceae were prevalent (Figure 1b). This suggests that Glomeraceae prefer neutral to slightly alkaline conditions [42], while Claroideoglomeraceae and Acaulosporaceae may be better adapted to lower pH and calcium environments [43,44]. Additionally, we observed a decrease in AMF community diversity in plots with high pH and high calcium content (Table 2). This reduction may result from soil environment alterations following the application of calcium-containing passivators, creating conditions more favorable for specific AMF taxa. These environmental changes may also affect Cd availability or enhance phosphorus fixation, thereby indirectly impacting certain AMF taxa’s survival and reducing overall AMF diversity [28,45]. Nevertheless, our study is limited by the absence of direct measurements of available Cd concentrations, preventing a deeper analysis of how pH indirectly influences AMF community composition. Despite this, our findings provide valuable regulatory targets for AMF-based Cd-contaminated paddy field remediation, warranting further verification through combined calcium and phosphorus addition with pH adjustment.

## 4. Materials and Methods

### 4.1. Study Area

The study area encompasses four counties and districts in Jiangxi Province: Wannian County (WN) in Shangrao City, Jinxi County (JX) in Fuzhou City, Nankang District (NK) in Ganzhou City, and Yushui District (XY) in Xinyu City. Within these areas, five plots located in the safe-utilization zones of Cd-contaminated paddy fields were selected for soil sampling. The region experiences a subtropical monsoon climate, The basic soil characteristics are red soil and paddy soil, with average annual temperatures between 16 and 20 °C and annual precipitation ranging from 1300 to 1900 mm. The soil Cd concentrations ranged from 0.1 to 1.5 mg/kg, and soil pH values ranged from 4.5 to 6.5.

### 4.2. Collection of Soil Samples

Soil sampling was carried out at the designated sites in July and August 2023. At each sampling site, five paddy plots (minimum area: 300 m^2^ per plot) were selected with a minimum spacing of 20 m between any two plots. In each paddy plot, five sampling points (at the four corners and the center of the field) were arranged. Surface debris was removed from the paddy soil layer and soil from the 0–20 cm plow layer was collected vertically. The collected soil samples from each point (~200 g) were thoroughly mixed, yielding a total of 1 kg of soil sample per plot. The larger particles along with fine plant roots were removed. The samples were immediately sealed in sterile pre-labeled bags and stored in an insulated container with dry ice. Transportation of soil samples to the laboratory was prioritized for immediate processing. Upon arrival, 50 g of each sample was stored at −80 °C for subsequent AMF community structure sequencing, while the remaining soil was air-dried for the assessment of physicochemical properties.

### 4.3. Determination of Soil Physicochemical Parameters

The soil samples underwent gradient digestion on a hot plate using a mixed acid solution composed of HNO_3_, HF, and HClO_4_ in a 5:2:1 (*v*/*v*) ratio. Subsequently, the volume was adjusted, and Cd content was determined using inductively coupled plasma mass spectrometry (ICP-MS, Thermo RQ, Waltham, MA, USA) [11]. Total phosphorus and calcium concentrations were measured using inductively coupled plasma optical emission spectrometry (ICP-OES, Thermo RQ, USA). To assess available phosphorous concentrations, the soil sample was extracted with an NH_4_F-HCl solution at a 1:10 (*w*/*v*) ratio, followed by centrifugation and analysis via the molybdenum–antimony colorimetric method [46]. Total nitrogen content was measured using a fully automated Kjeldahl nitrogen analyzer (ATN-300, Shanghai Hongji Instrument Equipment Co., Ltd., Shanghai, China) after digestion with H_2_SO_4_, distillation, and titration [47]. Alkali-hydrolyzable nitrogen was determined using the alkali-hydrolysis diffusion method, while soil organic matter was quantified using the potassium dichromate oxidation method. For pH measurement, a soil suspension was prepared at a 2.5:1 (*w*/*v*) soil-to-water ratio, agitated, allowed to settle for 30 min, and measured with a pH meter (FE28, METTLER TOLEDO, Columbus, OH, USA) [48]. To ensure data accuracy, this experiment strictly adheres to the SCI quality control system. The curve correlation coefficient (r^2^) must be equal to or greater than 0.995, and the concentration of the tested samples should fall within the linear range of the curve. Additionally, one full-process blank must be set for every ten samples, with the background value remaining below the detection limit.

### 4.4. Determination of AMF Community Structure

High-throughput sequencing technology was utilized to investigate changes in the structure of AMF communities. The total DNA from soil samples collected from each plot was extracted in strict accordance with the instructions provided by the E.Z.N.A.^®^ Soil DNA Kit (Omega, Mountain Lakes, NJ, USA). The concentration and purity of the extracted DNA were quantified using a NanoDrop2000 spectrophotometer (Thermo Scientific, Waltham, MA, USA) to ensure compliance with the requirements for subsequent sequencing procedures. Two rounds of nested PCR amplification were conducted on the 18S rDNA small subunit (SSU) of AMF, using the primer pairs AML1/AML2 [49] and AMV4.5NF/AMDGR [50], respectively. The purity of the amplified products was verified through electrophoresis, and upon confirmation, the sequencing of the amplified products was completed on the Illumina MiSeq platform (Illumina, San Diego, CA, USA). A minimum of 45,000 sequences were required per sample (sequences from each sample were rarefied to 45,829). The minimum sequencing depth per sequence was 200 bp, with an average of 215 bp. All the above experimental procedures were carried out by Shanghai Majorbio Bio-pharm Technology Co., Ltd. (Shanghai, China).

The raw sequencing data were processed to remove adapter sequences, and subsequently split and assembled using FLASH software (version 1.2.11) [51,52]. Following this, chimeric sequences were screened using UCHIME software (version 4.2.40). The sequences were clustered into operational taxonomic units (OTUs) at a 95% similarity level and annotated using UPARSE software (version 7.1). These were then compared against the MaarjAM [53] taxonomic database. The MaarjAM database is accessible at https://maarjam.ut.ee/ (accessed on 24 March 2024).

### 4.5. Statistical Analysis

One-way analysis of variance (ANOVA) was employed to evaluate differences in soil physicochemical properties and the α-diversity of AMF communities across five paddy field plots. The analysis began with rarefaction processing using the phyloseq package in R software (version 4.4.1), followed by Duncan’s test to analyze the variance in environmental factors and AMF community structure, thereby evaluating the significance of differences between various treatments and each indicator. A Venn diagram was created using the Venn Diagram plugin in Origin 2021 software to provide a more intuitive comparison of the differences in AMF community OTUs. To examine the correlation between AMF communities and environmental factors, Pearson correlation analysis in OmicShare Tools software (http://www.omicshare.com/tools, accessed on 13 September 2024) was utilized to generate a heatmap illustrating the relationship between soil nutrients and species richness. The AMF community composition among different plots was compared by calculating the distances between samples using the vegan package, sorting and reshaping the data with the dplyr and reshape2 packages, and visualizing the β-diversity and AMF community structure through non-metric multidimensional scaling (NMDS), redundancy analysis (RDA), and stacked bar charts using the ggplot2 package.

## 5. Conclusions

This research examined the influence of varying levels of Cd contamination in paddy fields on AMF community structure, elucidating the regulatory mechanisms through which Cd affects AMF distribution and diversity. The key findings are as follows:

The AMF families Claroideoglomeraceae and Glomeraceae were found to predominate in Cd-contaminated paddy fields. Notably, Claroideoglomeraceae showed significantly enhanced growth under Cd exposure, suggesting a high degree of Cd tolerance.

The relationship between Cd concentration and AMF communities exhibited a nonlinear pattern. At lower Cd concentrations, the growth of certain AMF taxa may be stimulated resulting in increased α-diversity. However, at higher Cd concentrations, selective pressure intensified, leading to the decline in Cd-sensitive taxa and the proliferation of tolerant dominant species, ultimately reducing overall AMF diversity.

The interplay between soil physicochemical properties and Cd influenced the AMF community composition. Elevated levels of available phosphorus were associated with reduced AMF diversity, whereas elevated calcium content and alkaline pH conditions favored the enrichment of Glomeraceae.

These findings establish a theoretical foundation for the application of AMF inoculation in the remediation of Cd-contaminated paddy fields. Nonetheless, additional research is required to validate the dose-dependent effects of Cd through gradient studies and to investigate the synergistic influence of multiple environmental factors in order to optimize AMF-based strategies for the remediation of Cd pollution in paddy fields.

## Figures and Tables

**Table 1:** Soil physical and chemical properties of different treatment sites.

Treatment	OM (g/kg)	AP (mg/kg)	TN (g/kg)	AN (mg/kg)	TP (mg/kg)	Ca (mg/kg)	Cd (mg/kg)	pH
JX (27.960° N, 116.834° E)	40.98 + 6.26 ab	15.44 + 5.80 b	2.53 + 0.33 a	0.03 + 0.01 a	560.0 + 157.65 ab	808.20 + 179.29 b	0.67 + 0.17 b	5.02 + 0.08 c
NK (25.718° N, 114.747° E)	26.48 + 3.66 d	14.61 + 13.45 b	1.71 + 0.18 c	0.02 + 0.01 a	589.2 + 136.40 ab	1168.4 + 1030.08 b	0.36 + 0.07 c	5.39 + 0.65 c
WN (28.772° N, 117.078° E)	35.98 + 2.80 bc	57.10 + 29.56 a	2.52 + 0.20 a	0.04 + 0.02 a	707.00 + 128.34 a	532.40 + 109.62 b	0.39 + 0.05 c	5.14 + 0.13 c
XY_1 (27.794° N, 114.994° E)	43.52 + 2.35 a	56.82 + 10.69 a	2.66 + 0.19 a	0.03 + 0.00 a	518.40 + 19.49 bc	2254.00 + 114.59 a	1.36 + 0.40 a	5.97 + 0.22 b
XY_5 (27.789° N, 115.000° E)	31.44 + 3.38 cd	16.90 + 5.79 b	2.17 + 0.25 b	0.03 + 0.01 a	399.40 + 60.58 c	2954.00 + 686.75 a	0.56 + 0.02 bc	6.86 + 0.23 a

Notes: OM, organic matter; AP, available phosphorus. TN, total nitrogen; AN, alkali-hydrolyzed nitrogen; TP, total phosphorus; Ca, total calcium; Cd, total cadmium. Different letters within the same row indicate significant differences among the treatments (*p* < 0.05).

**Table 2:** Analysis of AMF alpha diversity (*p* < 0.05). Different lowercase letters in the table indicate significant differences between different treatments (*p* < 0.05).

Treatment	Chao1 Index	ACE Index	Simpson Index	Shannon Index
JX	31.90 ± 9.75 a	32.09 ± 9.52 a	0.69 ± 0.07 a	2.24 ± 0.34 a
NK	31.19 ± 19.35 a	32.03 ± 19.64 a	0.65 ± 0.15 ab	2.19 ± 0.73 a
WN	11.50 ± 4.80 b	12.55 ± 6.02 b	0.47 ± 0.24 ab	1.25 ± 0.61 b
XY_1	25.67 ± 11.95 ab	26.07 ± 12.10 ab	0.53 ± 0.28 ab	1.79 ± 0.97 ab
XY_5	12.00 ± 3.54 b	12.44 ± 3.62 b	0.38 ± 0.20 b	1.12 ± 0.52 b

## Data Availability

The original contributions presented in this study are included in the article. Further inquiries can be directed to the corresponding authors.
